# Physician empowerment programme; a unique workshop for physician-managers of community clinics

**DOI:** 10.1186/s12909-016-0786-y

**Published:** 2016-10-14

**Authors:** Yafit Maza, Efrat Shechter, Neta Pur Eizenberg, Efrat Gortler Segev, Moshe Y. Flugelman

**Affiliations:** 1 Department of Human Resource Development Division, Clalit Health Services, Tel Aviv, Israel; 2 Department of Cardiovascular Medicine, Lady Davis Carmel Medical Center, and the Rappaport Faculty of Medicine, Technion IIT, 7 Michal Street, Haifa, 34362 Israel

**Keywords:** Faculty development, Medical education professionalism, Physician behaviour, Physician burnout, Physician empowerment

## Abstract

**Background:**

The physician manager role in the health care system is invaluable as they serve as role models and quality setters. The requirements from physician managers have become more demanding and the role less prestigious; yet burnout and its prevention in this group have received little attention. Physician leadership development programmes have generally dealt directly with skill and knowledge acquisition. The aim of this research was to evaluate an intensive workshop designed to modify attitudes and improve skills of physician-managers of community clinics, through focus on personal well-being and empowerment.

**Methods:**

Two hundred fifty six physicians affiliated with Clalit Health Services, the largest health maintenance organization in Israel, participated in 16 IMPACT courses during the years 2013–2015. The programme comprised five full days during a two-week period, including an overnight and follow-up meetings three and six weeks later. Theoretical knowledge, experiential learning, practical tools, deep personal exercises, and simulations were conveyed through individual and group work. Topics included: models of self-awareness, outcome thinking, determining a personal and organizational vision, and creating a personal approach to leadership. At the end of each course, and by email at 6 or more months after completion of the course, participants were asked to anonymously respond to closed questions (on a scale of 1–6) and an open question.

**Results:**

Mean scores for the contribution of IMPACT to participants’ role of physician manager were 5.3 at the end of the course, and 4.7 at 6 or more months later. Mean scores at 6 or more months were 5.0 regarding the contribution of the programme to personal development, 4.4 regarding satisfaction in the role of physician manager, and 4.6 regarding their coping with managerial dilemmas.

**Conclusion:**

A workshop that focused on personal growth and self-awareness increased physicians’ job satisfaction and their sense of managerial capability, coping with managerial dilemmas, and belonging to the organization.

## Background

The strain borne by physicians in the public health care system [1, 2], and particularly in primary care, has become well recognized [3, 4]. Burnout of physicians is a common phenomenon, across specialties and geographic regions [2, 3, 5, 6]. In the public health care system, heavy patient burden and insufficient numbers of physicians are reasons for physician burnout. In primary care, low interest among medical students, relatively low compensation, and increasing bureaucratization, and computerization of the health care system are additional reasons [7–9]. Physicians in the US were found to be more likely to have symptoms of burnout than other working adults, with primary care physicians demonstrating particularly high rates [6]. Burnout can result in dysfunction at multiple levels, including low empathy [10], compassion fatigue [4], reduced efficiency, (self-perceived) medical errors [11], and (self-reported) suboptimal patient care practices [12]. Such outcomes can further increase burnout, leading to a vicious cycle. The promotion of personal and professional physician wellbeing has become recognized for its merit in general [13], and particularly as a means of preventing physician burnout [14]. Contrasting with the accumulating research and interventions on the pervasiveness and prevention of physician burnout, the specific strain on clinical managers has received relatively little attention.

Physician managers have a pivotal role in health organizations as they set the medical standard, oversee quality programs and education, and serve as role models. The documentation of physician leadership development programmes over the years attests to the recognition of a need to address the skills and performance of physicians in managerial roles. Balint groups and other types of support groups, based on personal awareness and the sharing of meaningful work experiences, have been shown to help manage stress and to improve physician well-being and work satisfaction [15–19]. Mindfulness meditation, self-awareness exercises, and discussions about meaningful clinical experiences were shown to improve mindfulness and relaxation among physicians and to decrease assessed burnout [20, 21].

Clalit Health Services (CHS) provides community and hospital-based health care to 55 % of Israeli citizens (about 4.5 million individuals). Physician-managers are responsible for the 350 community clinics that are served by at least 3, and up to 15 physicians, and that include nursing staff, administrative staff, and pharmacies. All physician-managers take care of patients and have protected and paid time for management of their clinics. Physician-managers are selected for the position based on professional leadership and administrative capabilities. Their responsibilities include supervision of quality of medical work, continued education of physicians, solving staff and patient complaints and disputes, and the ongoing running of the clinic. Professional education for the management position includes workshops and organizational consultation on such topics as health care in the CHS system and in Israel, human resource management, and conflict management. Over the years the role of physician-manager in CHS became more demanding, while its status and perception among physicians waned. Decreased interest for this pivotal role led to difficulties in recruiting physicians to fill it. Similarly, a recent Canadian study based on an online survey of frontline clinical managers concluded that manager job strain decreases interest in filling the position, contributes to burnout, and reduces organizational commitment [22].

CHS established a committee that would seek ways to make the physician manager role more attractive. One of the decisions was the development and implementation of IMPACT, an intensive workshop in self-development and personal empowerment, to be offered exclusively to physician-managers. The rationale was that personal empowerment would increase well being, which would increase job satisfaction, strengthen leadership, and improve the performance of physician managers. This report investigates whether implementation of a programme that focused on personal well-being and empowerment had a positive effect on attitudes of physician managers to their position, and improved their skills in dealing with personal and professional problems, and their coping with managerial dilemmas. As such, we examined whether a programme that does not deal directly with skill and knowledge acquisition may modify learning, skills and attitudes, and even some behaviours; thus promoting change corresponding to levels 2 and 3 in the Kirkpatrick model (http://www.kirkpatrickpartners.com).

## Methods

During the years 2013–2015, 256 of the 350 physician managers of Clalit community clinics participated in one of 16 IMPACT courses. All participants received their regular payment for the 5 days of the workshop.

Participants in IMPACT were 140 men and 116 women. Their ages were: under 40 years (4 %), 41–50 years (34 %), 51–60 years (46 %), and over 60 years (16 %). Their years of experience as physicians in CHS were: 2–10 years (9 %), 11–20 years (40 %), 21–30 years (35 %) and 31–40 years (16 %).

Two moderators, Mati Harlev and Tal Bashan, with extensive experience as consultants, lecturers and group leaders in the fields of self empowerment and conscious leadership, developed the unique IMPACT programme, and implemented all the courses. The goal of IMPACT is to facilitate the realization of personal empowerment, and to provide tools for directing this power to private and professional aspects of life, and to conscious leadership. The rationale is that the key to enhancing personal growth and to taking control of forces that hinder such growth are to be found within a person. The process of identifying and addressing personal issues presents a mirror for growth in various realms. Increased awareness and responsibility in decision making, together with active choice, result in intentional and desired outcomes. Manifestations are expected in all aspects of life. The impetus for the project was the success of personal empowerment workshops in large organizations. Through IMPACT, the top management of CHS aimed to convey a message of particular investment in the role of physician manager.

IMPACT is carefully structured. The approach is intense, introspective and simple. Components are theoretical knowledge, experiential learning, practical tools, deep personal exercises and simulations. Learning is on an individual level, in dyads, and through group intimacy. Participants interact with other physician-managers within a personal, rather than professional context. During the course of the workshop, participants are afforded the opportunity of detaching themselves from their affiliated organization, from management, from work, from mails and telephones, from the worries of daily life, from home and family, from the masks they adorn in their personal and professional lives, and from their “false” selves. They have the opportunity to connect with their inner selves, personal power, inner drives, and their capability of choice in personal and managerial behaviour; and to gain a new sense of belonging to their affiliated organization.

Table 1 presents the topics and main contents of the five days of the IMPACT programme, which are held during a two-week period. During the second week, a 36 h retreat takes place at a secluded high standard guest house; telephones and televisions are made unavailable. The first day of the retreat ends at 10 PM, at which time the participants return to their rooms with personal assignments related to the day’s events. The following morning is dedicated to special group and solo activities in an outdoor and natural setting. On the fifth and last day of each course, senior managers and regional managers of CHS visit; they express their commitment to the project and to the physician-managers, and listen to feedback. Two follow-up meetings are held at 3 and 6 week intervals after the workshop.

**Table 1:** Summary of the topics and content of the 5 days of IMPACT

Meeting	Topic	Main content
WEEK 1 Monday	Introduction and Acquaintance	Greetings, presentation of the program, group acquaintance, setting expectations
	Perception of role	Recognition of the unconscious perception of the managerial role
Tuesday	Personal awareness and personal vision	Acquaintance and experience with different models of self-awareness: Distinguishing and choosing life approaches: reacting / acting / creating Understanding and taking control of the processing of events: from incident occurrence, to interpretation, to elicited emotion, to possible reactions Identifying behavioral and emotional patterns, and examination of their fit with an individual perception of the managerial role and with a personal and organizational vision
	Outcome thinking	Developing outcome thinking Defining desired outcomes and developing patterns of thinking to realize these outcomes. Identification of undesired outcomes and examination of thinking patterns that educe them.
WEEK 2 Sunday-Monday	Tools to achieve desired goals based on personal qualities and abilities	Field workshop including an overnight stay. Applying personal qualities to achieve desired results in personal and professional realms Developing innovative thinking and discovering new means of coping Analysis of managerial dilemmas, using novel thinking to creatively change situations. Solo exercise – participants are sent for a solo exercise in the field. This exercise is a dramatic experience, intense, and meaningful, which promotes deep understanding of the ways by which habitual patterns obstruct self-realization and the achievement of desired outcomes
	Personal profile with insights on leadership	Processing of the solo exercise and the insights gained thus far. Identification and clarification of factors that cause personal fatigue, and acquisition of tools to deal with them Creation of a personal leadership profile
Tuesday	Identifying personal style and approach	Integrative exercise to summarize the course and to catalyze application in personal and professional realms
	Conclusions and feedback	Individual and group feedback and conclusions

Questionnaires to be answered anonymously were distributed to participants at the end of each course, and by email at 6 or more months after completion of the course. Closed questions addressed the physician managers’ satisfaction with the programme and their assessment of its contribution to their personal lives, to their role of physician managers, and to their coping with managerial dilemmas. Responses were on a scale of 1–6 (1 – not at all to 6 – very much / very highly). Open questions invited the participants to express themselves more freely regarding their experience in the programme and its impact in their personal and professional realms.

## Results

Response rates were 231/256 (90 %) and 108/256 (42 %) for the respective questionnaires (Table 2). Mean ratings of overall satisfaction were 5.7 and 5.4, respectively (on a scale of 1–6); and mean assessments of expectations of the contribution of the course to the role of physician manager were 5.3 and 4.7, respectively. To the long term questionnaire, 75 % responded that the course contributed highly or very highly to their personal development. The particular aspects that received the highest ratings after 6 or more months were contributions to self awareness and to the awareness of forces that drive and hinder actions. The aspect that received the lowest rating was the contribution of the course to increasing satisfaction in the role of physician manager; nonetheless, 44 % of the respondents rated the contribution of the course high or very high, and 39 % rated it as moderately high. Regarding the importance that CHS attributes to the development of physician managers, as reflected in implementation of the course, 91 % responded “high” or “very high”. Compared with the evaluation immediately at the end of the course, a certain decrease in scores was noticeable six or more months later. On the follow-up questionnaire, 53 % rated 5 or 6 (on a scale of 6) their improved coping with managerial dilemmas.

**Table 2:** Responses of participants in a pilot and in 15 courses to a questionnaire distributed at least 6 months after the course and a questionnaire distributed at the end of the course

Question	Course end	At least 6 months after course				
	Mean	5, 6	4	3	1,2	Mean
Evaluation of the program						
How satisfied were you with the program in general?	5.7	84 %	15 %	1 %	-	5.4
How much did the program contribute to your personal development?		75 %	18 %	7 %	-	5.0
How much did (do you expect) the program (to) contribute to your role as physician manager?	5.3	64 %	24 %	10 %	1 %	4.7
Satisfaction in your role as a physician manager		44 %	39 %	14 %	4 %	4.4
Sense of engagement to CHS		64 %	21 %	9 %	6 %	4.7
Opinion: To what degree does the implementation of this type of program reflect the importance that CHS attributes to the development of physician managers?	5.8	91 %	8 %	2 %	-	5.5
Assessment of modifications in attitudes and skills						
Awareness and deep understanding of the forces that drive you	5.5	83 %	14 %	3 %	-	5.2
Awareness and deep understanding of the forces that hinder you	5.4	79 %	16 %	6 %	-	5.1
Ability to maintain internal balance	5.2	71 %	20 %	8 %	-	5.0
Sense of well being		62 %	23 %	11 %	4 %	4.7
Elimination of automatic patterns	5.3	65 %	25 %	10 %	-	4.9
Expansion of your extent of influence	5.2	62 %	29 %	8 %	1 %	4.7
Self awareness	5.6	81 %	14 %	5 %	-	5.3
Interpersonal communication		73 %	10 %	14 %	4 %	4.8
Personal leadership ability	5.2	71 %	20 %	7 %	1 %	4.9
Assessment of modifications in behaviors						
Pro-activity in management	5.2	70 %	18 %	12 %	-	4.9
Coping with managerial dilemmas		53 %	35 %	11 %	1 %	4.6

Responses were on a 6 point scale: from 0 (not at all) to 6 (very much / very highly)

In open questions, the participants reported that IMPACT contributed to their understanding of themselves and to their interactions with others in their personal and professional realms. Many emphasized the uniqueness of the workshop, remarking on its experiential quality and the substantial repercussions realized in diverse life situations. They expressed great appreciation for the opportunity to detach physically and mentally from work and to focus on themselves, their personal capabilities, desires, and fulfillment. In addition to the specific aspects referred to in the closed questionnaire, self-confidence was mentioned as an area that improved following the course. Though managerial issues and the healthcare organization were not directly addressed in the workshop, participants wrote that they received tools that contributed to their managerial role, specifically in planning, coping with situations, and interactions with patients and employees in their clinics. Moreover, participants stated that IMPACT contributed considerably to their roles as managers and enhanced their feeling of belonging to the organization. They found it helpful to share with others who had common problems. Many expressed interest in continued meetings, and some said that they themselves had already organized follow-up meetings for the participants in their course.

## Discussion

Following the Kirkpatrick model, this research demonstrated that IMPACT primarily modified learning, skills and attitudes (level 2); yet also modified some behaviours (level 3). Not surprisingly, the scores on behavioural improvement were slightly lower than those on skill improvement.

From the participants’ written and oral responses, the perception of CHS management is that IMPACT has achieved its goal of empowering conscious leadership through self-development. The implementation of a workshop for all physician managers in a large organization created a shared experience, which enhanced the potential of changing the perception of the role within the organization. From the viewpoint of CHS, embarking on such an initiative required organizational courage. Substantial resources were invested, without any known precedence, in Israel or around the world, for such an extensive programme in self-development and empowerment for physicians. Success of the first course motivated CHS management to offer IMPACT to all physician managers.

The participation of 73 % of physician managers in IMPACT is a noteworthy achievement of the CHS organization. The 90 % response rate at the end of the course is high. However, the response rate of 42 % at 6 months is a limitation of this study. We have no information as to whether this low response was due to physicians not bothering to fill the email questionnaire or to another reason. Moreover, the actual effect of IMPACT on the perception of the role of physician-managers in CHS cannot be assessed because during the period of its implementation, other changes occurred, such as an increase in financial compensation for the position. The effect of the workshop on actual changes in work performance was not within the scope of this report.

A recently published systematic review identified 35 studies that reported on physician leadership development programmes [23]. The majority of programmes focused on skills training and technical and conceptual knowledge. Personal growth and self-awareness did not comprise central aspects of any of the studies. Other recent publications have described a number of physician training programs based on mindfulness [24–26]. A randomized trial showed that 19 biweekly facilitated physician discussion groups involving mindfulness, reflection, and shared experience increased empowerment and engagement at work and decreased depersonalization and burnout [25]. Coaching has also been proposed as a means of increasing self-awareness and of aligning personal values with professional duties, and thus decreasing physician burnout [27]. Training in managerial skills such as budget management, business development, and staff supervision can undoubtedly improve performance of physician managers but are beyond the scope of the current report.

The Israeli national health care system provided fertile territory for the IMPACT initiative. As a large healthcare organization, CHS was able to release physician managers from their work responsibilities and afford them the opportunity to participate in an intensive workshop. A unique feature of IMPACT is the dedicated time (5 full days, including an overnight, during a two-week period, and 2 follow-up days). Even interventions for physicians that entail 2.5 h weekly sessions over an eight week period have been considered intensive [21, 26]. Evidently, the fact that the participants received complete pay during the full days of the workshop contributed to their positive attitude to the organization. Along this line, a randomized trial of an intervention implemented by a single medical center department showed that paid time (90 % salary) in itself improved outcomes in a control group, though less than in the intervention group [25]. While many health care organizations may not have the resources to implement such a large-scale programme as described herein, we believe that the approach and focus of IMPACT may be generalizable also to smaller scale programmes. The essence of IMPACT is placing personal development at the core of an initiative for physician-managers.

## Conclusions

A workshop that focused on personal well-being and empowerment contributed positively to participants’ sense of their managerial capability, evidently because it dealt directly with the person who is a manager and not with the manager as a role. Future assessments of job satisfaction and burnout among the participants will reveal long term effects of the programme on attitudes toward their work, acquired skills and behavioural changes.
